# Comparative analysis of chromatin landscape in regulatory regions of human housekeeping and tissue specific genes

**DOI:** 10.1186/1471-2105-6-126

**Published:** 2005-05-26

**Authors:** Mythily Ganapathi, Pragya Srivastava, Sushanta Kumar Das Sutar, Kaushal Kumar, Dipayan Dasgupta, Gajinder Pal Singh, Vani Brahmachari, Samir K Brahmachari

**Affiliations:** 1 Dr. B. R. Ambedkar Centre for Biomedical Research, University of Delhi, Delhi-110007, India; 2 Institute of Genomics and Integrative Biology (CSIR), Mall Road, Delhi -110007, India

## Abstract

**Background:**

Global regulatory mechanisms involving chromatin assembly and remodelling in the promoter regions of genes is implicated in eukaryotic transcription control especially for genes subjected to spatial and temporal regulation. The potential to utilise global regulatory mechanisms for controlling gene expression might depend upon the architecture of the chromatin in and around the gene. *In-silico *analysis can yield important insights into this aspect, facilitating comparison of two or more classes of genes comprising of a large number of genes within each group.

**Results:**

In the present study, we carried out a comparative analysis of chromatin characteristics in terms of the scaffold/matrix attachment regions, nucleosome formation potential and the occurrence of repetitive sequences, in the upstream regulatory regions of housekeeping and tissue specific genes. Our data show that putative scaffold/matrix attachment regions are more abundant and nucleosome formation potential is higher in the 5' regions of tissue specific genes as compared to the housekeeping genes.

**Conclusion:**

The differences in the chromatin features between the two groups of genes indicate the involvement of chromatin organisation in the control of gene expression. The presence of global regulatory mechanisms mediated through chromatin organisation can decrease the burden of invoking gene specific regulators for maintenance of the active/silenced state of gene expression. This could partially explain the lower number of genes estimated in the human genome.

## Background

Eukaryotic gene transcription is largely known to be orchestrated by protein factors like activators, co-activators and co-repressors [1]. However, nucleosomal organisation, non-passive structural scaffolds and global structure of chromatin are increasingly being recognised as major players in the regulation of gene expression. The ability of sequences to position nucleosomes and to be anchored to the nuclear matrix to provide a spatial context for regulation of expression are measurable parameters that may influence the interactions with transcription machinery [2,3]. This level of regulation may be distinctly different for genes whose expression is constitutive in comparison to genes that exhibit tissue specific expression. The latter would demand an open chromatin configuration in certain tissues and repressive organisation in others. In this study, we examined whether the potential to utilise global regulatory mechanisms to control gene expression through chromatin organisation varies between housekeeping and tissue specific genes (Hkg and Tsg respectively) by virtue of their organisation. An *in-silico *comparison of chromatin related organisational differences in the 5' and 3' regulatory regions of housekeeping and tissue specific genes was carried out to shed light in this direction.

## Results and discussion

Chromatin landscape of a region plays a major role in determining and modulating the expression status of its neighbouring genes [4]. The role played by chromatin in the 5' regulatory regions of genes in transcriptional regulation has been extensively studied [5,6]. In the present study, we have taken 2 distinct sets of genes differing predominantly in their spatial expression aspect, namely, housekeeping and tissue specific, to understand the various attributes of the regulatory role played by chromatin organisation in the 5' region.

### Analysis of scaffold/matrix associated sequences

Scaffold/matrix attachment regions (S/MARs) are defined as sequences, which can attach themselves to the nuclear matrix and hence help in the formation of independent chromatin loops [7]. Transcriptional regulation of gene expression is known to involve formation of dynamic chromatin loops mediated by S/MAR attachment to the nuclear matrix [3]. The attachment of a DNA sequence to the matrix will place the neighbouring genes in proximity of the transcription factors. The abundance of S/MARs in the 5' *cis*-regulatory regions of genes further demonstrates their role in transcriptional regulation [8]. We have analysed the predicted S/MAR sites in the 5' and 3' flanking regions of human Hkg and Tsg (Table 1). We used MAR Finder (new version) and ChrClass programs for predicting S/MAR binding sites in the sequences (Table 1). Glazko *et al *have classified 5' flanking regions up to 1500 bp of human tissue specific genes as an out-group, assuming that these regions have no significant association with S/MAR binding [7]. On the contrary, our study reveals that S/MAR binding sequences are enriched in 5' regulatory regions of Tsg in comparison to the Hkg. The common predictions of both the programs were taken for the analysis. This data indicates a significant enrichment of S/MAR binding sequences in the 5' flanking regions of Tsg and depletion of S/MARs in the 3' Hkg regions as compared to Tsg. Chi-square test was applied for both 5' and 3' region S/MAR predictions of Hkg and Tsg, to ascertain whether the distributions are significantly different. The chi-square value of 11.37 (df = 1) and *P*-value ≤ 0.001 obtained for the distribution of S/MARs in 5' regions of Hkg and Tsg indicate a significant difference in the distribution of S/MAR elements between the two sets. Similarly, for the distribution of S/MARs in 3' regions of Hkg and Tsg the chi-square value of 5.033 (df = 1) and *P*-value of ≤ 0.025 show that the Hkg 3' regions are significantly depleted of S/MARs as compared to Tsg.

The observation that the 5' regulatory regions of Hkg are less enriched in S/MARs in comparison with Tsg might be related to the distribution of housekeeping genes in the genome. Housekeeping genes cluster in chromosomes and therefore, they often would be present in distinct chromatin domains along with housekeeping genes that have a co-ordinated expression [9,10]. The data showing preferential absence of S/MARs in the 3' regions in Hkg further lend support to this hypothesis. On the other hand, tissue specific genes are known to be dispersed in gene dense as well as heterochromatic regions [9,11]. It may be necessary for them to shield themselves against the effects of positive and negative *cis*-acting elements of adjacent regions in order to maintain tissue specific expression profile. In this context, the boundary elements or the insulator model has been proposed earlier [11]. S/MARs function as boundary elements and their co-localisation with insulators such as the Drosophila gypsy element is also reported [12,13]. They also function as boundary elements in *in vitro *systems by shielding away the position effect [14]. Some earlier reports have suggested a role for S/MARs in maintaining tissue specific gene expression [15]. More recently, the 5'-HS4 chicken-globin insulator is known to have a CTCF protein binding dependent matrix association [16]. Hence, the over representation of S/MARs seen in Tsg set might possibly be associated with a boundary element function.

Our results on the prediction performance of the programs have been quite different from the previous reports [7]. We find that MAR Finder (an under predictor) predicts more number of S/MAR regions in our dataset in comparison to ChrClass program (an over predictor) [7]. This may be attributed to the use of the advanced version of MAR Finder in our study wherein, new parameters/features have been added in the form of the "New MAR Rules" option.

### Analysis of nucleosomal organisation

The primary template for local and global changes in the chromatin structure of a chromosome is the nucleosomal unit [4]. Chromatin structure and nucleosomal organisation over the promoter regions play a major role in regulation of expression of downstream gene(s) [6,17]. The nucleosome distribution would depend upon the occurrence of nucleosome destabilising elements as well as nucleosome forming sequences. We have analysed both these parameters in our study.

### Nucleosome destabilising elements

Nucleosome destabilising/excluding elements such as poly (dA.dT) and (CCGNN)_n _in promoter regions have been implicated in maintaining constitutive gene expression [18-21]. At the functional level, it is known that poly (dA.dT) elements increase the accessibility of promoters of HIS3, URA3 and Ilv1 in yeast to the cognate transcription factor [18]. With the increasing length of poly (dA.dT) repeat, the availability of the sequences to transcription factors improves and similarly, with increasing lengths, the propensity to exclude nucleosomes increases for (CCGNN)_n _sequence motif as demonstrated in yeast and mammalian systems [19-21]. It has been demonstrated that (CCGNN)_n _sequences promote meiotic recombination and activated HIS4 expression by generating open chromatin [22].

We hypothesised that the differential distribution of nucleosome exclusion elements might be one of the mechanisms involved in maintaining distinct nucleosomal organisation of the housekeeping and tissue specific genes. The frequency of pure poly (dA.dT) stretches >10 bp and (CCGNN)_2–5 _in the 2000 bp 5' *cis*-regulatory regions of human Hkg and Tsg(s) were analysed. A significant enrichment of poly (dA.dT) elements in the upstream regions of Hkg is seen in comparison to Tsg (Table 2). The *t*-test for the difference in distribution of poly (dA.dT) stretches (>10 bp) between Hkg and Tsg show significant *P*-values in the different lengths of the stretches examined.

In Hkg, 670 repeats of (CCGNN)_2–5 _were detected as against 430 in Tsg. (CCGNN)_2 _was the most prevalent repeat unit and uninterrupted repeat units (>5 mers) were not found in the sequence sets. Although shorter repeat units (2–5 mers) have not been studied for nucleosome exclusion, they might play a role in destabilising the histone octamer [20]. Further, many of them form a part of longer interrupted stretches. The *t*-test for difference in distribution of (CCGNN)_2–5 _between Hkg and Tsg shows a significant *P*-value of 1.71E-06.

### Nucleosome formation potential scores and expression level of genes

Using Recon, Levitsky *et al *(2001) have examined the nucleosome formation potential of 3 classes of human genes namely, Hkg, Tsg and widely expressed genes that differ in their spatial expression status [2]. Their report, based on a small sample size of around 200 genes shows the difference in the nucleosome formation potential between these 3 classes of genes in the upstream 50 bp from the transcription start site. In this study, we examined the nucleosome formation potential values in upstream 2000 bp of 5' regions of Hkg and Tsg and their correlation with gene expression levels with the complete set of 1083 genes.

**Figure 1:**
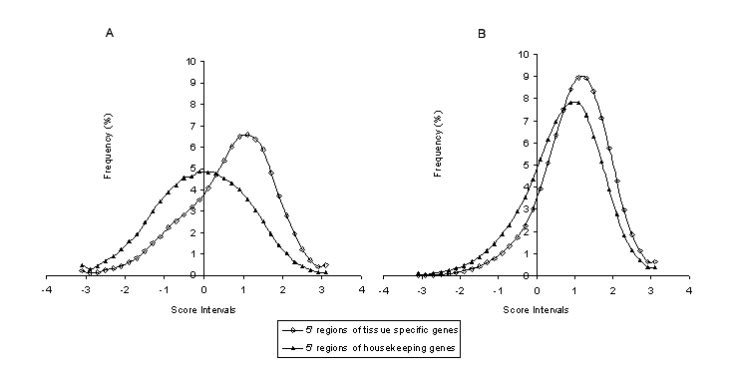
**Nucleosome formation potential score distributions for 5' regions of housekeeping and tissue specific genes**. The 5' sequences of human housekeeping and tissue specific genes were analysed by Recon for distribution of nucleosome formation potential scores. Frequency distribution histograms were plotted for scores in various intervals (range -3.2 to +3.2). (A) and (B) show the distribution of nucleosome formation potential scores at 400 and 2000 bp upstream from the gene start site respectively. Nucleosomal density is significantly lower for housekeeping genes as compared to tissue specific ones, in regions close to the gene start site.

**Figure 2:**
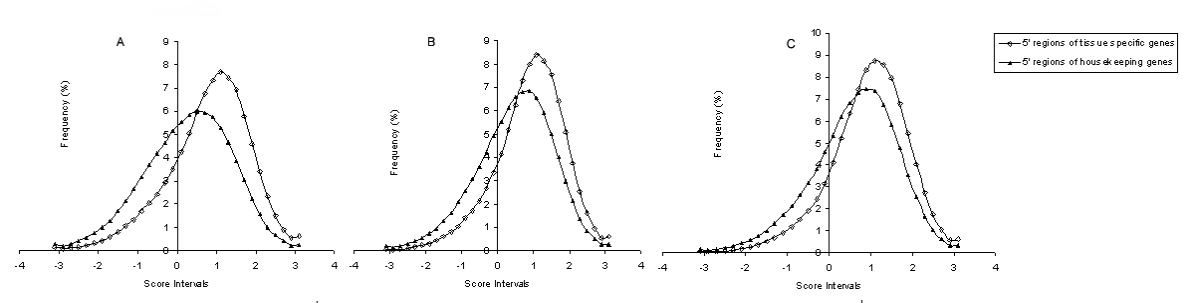
**Nucleosome formation potential score distributions for 5' regions at different positions from the gene start site in housekeeping and tissue specific genes**. The 5' regions of 800, 1200 and 1600 bp from the gene start site of housekeeping and tissue specific genes were taken for the analysis. Frequency distribution histograms were plotted for Recon scores in various intervals (range -3.2 to +3.2). (A), (B) and (C) show the distribution of nucleosome formation potential scores at 800, 1200 and 1600 bp upstream from the gene start site respectively. As we move upstream from the gene start site, the difference in the nucleosome formation potentials between housekeeping and tissue specific genes gradually fades away.

The Tsg and Hkg sequences show a considerable difference in their nucleosome formation potential scores over an extended upstream region of 2000 bp (Figures 1 and 2). The Tsg region is enriched in nucleosome formation potential scores (peak at 1) in all upstream positions analysed (till 2000 bp). For Hkg, the distribution seems to be shifted towards the negative scores at 400 bp region and this shift diminishes gradually as we move further upstream to finally peak at 1 in 2000 bp upstream region (Figure 1). *t*-test was applied to ascertain the difference in distribution of Recon scores between Hkg and Tsg (Table 3). The resultant *P*-values in various intervals of relevance (0.8 to 1, 1 to 1.2, -0.8 to -1 and -1 to -1.2) reflect that the scores in the upstream 400 bp from the gene start site show the maximum difference in all the intervals and at 2000 bp, the difference gradually fades away in intervals 0.8 to 1 and 1 to 1.2 (Table 3).

A correlation analysis between nucleosome formation potential and expression levels was carried out considering the Recon scores at upstream 400bp region, where the *P*-values reflect the largest difference and the log_10 _values of expression levels were taken as inputs ["see Additional file 1"]. Initially, we analysed the gross dependence of total expression levels on nucleosome potential in the upstream regions of the two sets of genes (Table 4). In all the four intervals, no correlation is seen, indicating that chromatin plays an insignificant role in global modulation of levels of expression in these two sets of genes. These results are similar to that observed in case of *Saccharomyces cerevisae *whole genome analysis (unpublished results).

Further, we refined the analysis to examine the correlation, if any, between nucleosome formation potential in upstream regions and extreme expression levels of genes. The Hkg and Tsg groups were further categorised separately into high and low expression level groups as described under "Methods" section and their correlation with the nucleosome formation potential was analysed (Table 5). The high and low expression genes of Hkg show a low negative correlation with scores in intervals 0.8 to 1.0 and 1 to 1.2 and a low positive correlation with scores in intervals -1.2 to -1 and -1.0 to -0.8. In Tsg, except in one interval, there was no valid correlation seen. This solitary value was not considered since the correlation coefficients in other intervals didn't reflect this trend.

Our data restates that chromatin in 5' region plays a major role in determining the ubiquitous or restricted tissue expression of a gene as shown by Levitsky *et al *(2001) [2]. The abundance of nucleosome exclusion elements in Hkg 5' regions and the low Recon scores reflect their poor preference for nucleosome assembly. The expression analysis suggests that although chromatin plays a role in bringing about extreme variations of gene expression levels in certain classes of genes such as the housekeeping genes, the relation is not linearly correlated with the total, wider range of expression levels. It is possible that nucleosomes might be involved in fine-tuning of expression levels that may escape our attention, since the difference in the range of expression considered is fairly large. The difference detected in nucleosome formation potential between the two sets might reflect the accessibility to basal transcription factors for Hkg and gene/tissue specific transcription factors for Tsg, considering the difference in spatial and temporal expression patterns of the two groups.

### Analysis of repetitive sequences

Repetitive sequences are implicated in chromatin organisation and heterochromatinisation [23-25]. They are differentially enriched in various functional categories of genes and are predicted to play an important role in gene regulation [24,26]. We analysed the distribution of various repeat classes in the 5' regions of Hkg and Tsg using RepeatMasker software. The total repeat content in Hkg regions is seen to be more than in Tsg regions. As reported earlier, our data shows enrichment of SINES (Alu) in comparison to other classes of repetitive sequences in both the sets [24]. Further, the 5' sequences of Hkg are more enriched in Alu sequences in comparison to those of Tsg regions (Table 6). The difference in the distribution of Alu repeats in the two classes of sequences was determined by applying *t*-test for the number of repeats and the repeat content in terms of length in base pairs in each sequence set (Table 7). The low total repeat content seen in Tsg upstream regions lends support to the hypothesis that condensed chromatin disfavours transposable element insertions in comparison to open chromatin (Hkg promoters)[27].

Genes with high expression levels are clustered in genomic regions known as ridges. These gene rich regions also have high (G+C) content, SINES and genes with short introns [9]. Eisenberg and Levanon [28] have reported the presence of significantly shorter introns and an overall compact gene structure in Hkg as compared to non-Hkg [28]. We have used the gene list provided by Eisenberg and Levanon [28] for our analysis. The enrichment of SINES in the 5' regions of Hkg suggests that Hkg might be localised in the ridge regions of the genome. More recently, it has been suggested that the contrasting attributes of gene compactness, GC content and the length of the intronic and intergenic sequences in Hkg and Tsg might be involved in chromatin mediated regulation for maintaining distinct expression patterns in the gene sets [29]. Recently, Alu elements have been shown to house transcription factor binding sites and the presence of such regulatory elements might influence the chromatin structure and gene expression [30].

**Figure 3:**
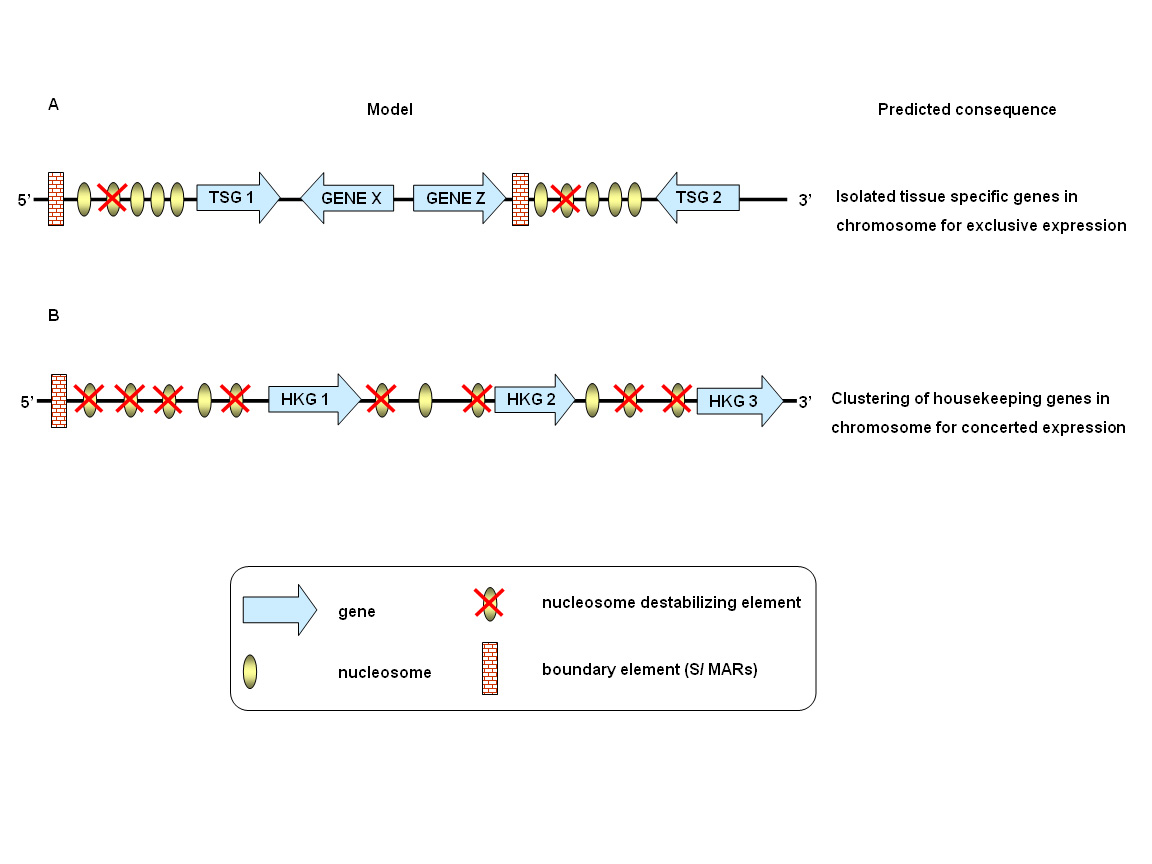
**A model for chromatin landscape in 5' regions of tissue specific and housekeeping genes**. (A) depicts the repressive role of chromatin in maintaining tissue specific gene expression profiles in a chromosome. The chromatin organisation in the 5' regions of Tsg1 and Tsg2, two different tissue specific genes dispersed in the chromosome is shown. Nucleosome formation potentials and S/MARs – the boundary elements, are enriched in their upstream regions and might play a major role in facilitating tissue specific expression. This is likely to be a local effect since neighbouring genes might have a different expression pattern. (B) depicts the chromatin organisation in the 5' regions of Hkg1, Hkg2 and Hkg3, three housekeeping genes clustered in the chromosome. The presence of low nucleosome formation potential regions and enrichment of nucleosome destabilising elements ensure an open chromatin configuration in this domain. As Hkg generally cluster together, they are depleted in S/MARs relative to tissue specific genes as shown in the present analysis by the significant absence of predicted S/MARs in both 5' and 3' regions of housekeeping genes as compared to tissue specific genes.

The paradigm for regulation of gene expression in human tissues has shifted the focus from involvement of a battery of transcription regulators to global regulatory mechanisms [31]. These mechanisms have also gained significance in the context of the low estimates of gene numbers in the human genome [32]. It is in this framework that we have analysed the chromatin characteristics of two groups of genes, one that needs almost a continuous and ubiquitous expression and another demanding tissue specific regulation. It had been predicted that the nucleosomal density in a chromatin domain and the buffering of supercoiling waves by repetitive DNA will play a major role in establishing coordinated gene regulation in a domain in the context of the relevance of maintenance of repetitive sequences during evolution [[25,33], and [34]]. A recent report also infers the role of chromatin-mediated mechanisms in the differential gene expression patterns seen in housekeeping and tissue specific genes [29]. Our data and analyses lend support to these hypotheses (Figure 3). Another recent report, which addresses the chromatin architecture of the human genome, provides experimental evidence that open chromatin correlates with high gene density regions but not with gene expression [35]. This data further supports our *in-silico *observations and strengthens the domain concept for concerted expression of clustered genes. The role of nucleosome formation potential is apparent from the present analysis in both the housekeeping genes as well as tissue specific genes but with an opposing correlation. Housekeeping genes apparently discourage nucleosome formation to match their expression profile in space and time by ensuring accessibility to transcription machinery. In addition, they also show a significant enrichment in poly (dA.dT) stretches, which are known to destabilise nucleosomes. On the other hand, the tissue specific genes show higher scores for nucleosome formation potential through which they perhaps provide selective accessibility to the transcriptional machinery. Further, our analysis suggests that tissue specific genes resort to additional global regulatory features such as matrix association, which would facilitate maintenance of functionally distinct domains to insulate themselves from both silencing and activating regulatory influence of adjacent domains. The differential distribution of repetitive sequences in housekeeping and tissue specific genes might also play an important role in maintaining distinct chromatin landscape over these regions.

## Conclusion

We have demonstrated that the regulatory regions of housekeeping and tissue specific genes have differential chromatin architecture with respect to S/MAR binding, nucleosome positioning potential and repetitive sequences. This has potential implications for regulation of gene expression in eukaryotic genomes.

## Methods

In this study, the 5' and 3' flanking regions of genes were analysed for various attributes of chromatin organisation. The list of human housekeeping genes (Hkg) was retrieved from [28,36]. 532 genes have been categorised as housekeeping because of their ubiquitous and high expression levels in 47 tissues. The list and expression levels of the human tissue specific genes were obtained from Eli Eisenberg (personal communication). 566 genes expressed in only a single tissue were taken as tissue specific genes (Tsg) and analysed. We could unambiguously retrieve sequences of 525 Hkg and 558 Tsg from human genome build 33 (NCBI). Approximately, 2000 bp of the 5' and 3' regions from each of these genes were taken for analysis.

### Scaffold/matrix associated regions (S/MAR) analysis

MAR Finder was used for prediction of S/MAR regions [37,38]. All the default options and the "New MAR Rules" were selected for predicting S/MARs. ChrClass program was used for S/MAR prediction [39,40].

### Nucleosome organisation and gene expression correlation analysis

The upstream regions (2000 bp) were scanned for nucleosome exclusion elements [18,20] – poly (dA.dT) pure stretches of >10 bp length and [5' (CCGNN) 3']_2–5 _using in-house programs. Recon was used for evaluating nucleosome formation potential in the sequences [2,41]. The score outputs of the 5' regions were categorised in frequency intervals of 0.2 with a range from -3.2 to +3.2. The Recon scores around +1 and -1 imply strong nucleosome formation and exclusion potentials respectively. The scores in the four intervals of relevance (0.8 to 1, 1 to 1.2, -0.8 to -1 and -1 to -1.2) were taken for all the analyses. Since the promoter region information was not retrieved for these genes, the 2000 bp upstream region from the gene start site was split into 400, 800, 1200 & 1600 bp and analysed.

The Recon scores at 400 bp were used to draw correlation between the nucleosome formation potential and expression levels in the two sets of genes. In each sequence set, genes with expression levels <500 and >5000 affymetrix expression units were classified as low and high expression genes respectively. We considered a minimum ten fold difference in the expression levels of genes as a relevant criterion for classifying them as high and low expression genes. In Hkg, this criterion yielded 33 low expression and 35 high expression genes. In Tsg, we categorised 416 low expression genes and 24 high expression genes.

### Repetitive sequence analysis

RepeatMasker version: 20040306-web was used to calculate the repeat content in 2000 bp upstream sequences of the two groups of genes [42].

## List of abbreviations

S/MAR: scaffold/matrix attachment regions

Hkg: housekeeping genes

Tsg: tissue specific genes

df: degree of freedom

## Authors' contributions

MG, VB and SKB contributed to the study design, analyses and in drafting the manuscript. MG, PS, SKDS, DD, KK and GP were involved in the data retrieval and analyses. All authors read and approved the final manuscript.

## Supplementary Material

Additional File 1 gene list and expression levels of housekeeping genes, gene list and expression levels of tissue specific genes. 'supplementaryfile1.xls' contains the list of housekeeping and tissue specific genes and their expression levels used for the analysis. Click here for file

## Figures and Tables

**Table 1:** Distribution of putative S/MARs in housekeeping and tissue specific genes.

	Putative S/MARs in 5' regions (%)*		Putative S/MARs in 3' regions (%)*	
	
**Prediction scheme**	**Hkg**^#^	**Tsg**^§^	**Hkg**^#^	**Tsg**^§^
presence of S/MAR	26.1	34.1	19.1	20.6
absence of S/MAR	26.1	19.2	34.5	25.1

^#^Housekeeping genes, ^§^Tissue specific genes,

* ChrClass and MAR Finder programs were used for prediction of S/MARs in housekeeping and tissue specific genes regulatory regions (5' & 3' regions – 2000 bp each). The common predictions of both the programs were used for the analysis. The data is represented as percentage of genes with predicted S/MARs in 5' and 3' regions of 525 housekeeping and 532 tissue specific genes.

**Table 2:** Distribution of poly (dA.dT) repeats of various lengths in the 5' upstream regions of housekeeping and tissue specific genes.

**Poly (dA.dT) stretch (bp)**	**No. of repeat stretches in the two classes**		**No. of genes with repeats in 5' region (%)**		
		
	**Hkg**^#^	Tsg*	**Hkg**^#^	Tsg*	^§^***P*-value**
>10	443	345	268 (51.0)	240 (43.0)	1.31E-04
>11	381	297	243 (46.3)	214 (38.4)	3.25E-04
>12	339	248	226 (43.1)	184 (33.0)	6.10E-05
>13	295	207	209 (39.8)	156 (28.0)	4.29E-05
>14	251	168	188 (35.8)	128 (22.9)	2.77E-05
>15	209	140	164 (31.2)	111 (19.9)	8.83E-05
>16	180	116	146 (27.8)	99 (17.7)	7.58E-05
>17	155	103	134 (25.5)	88 (15.8)	2.42E-04
>18	138	79	120 (22.9)	71 (12.7)	2.23E-05
>19	112	66	101 (19.2)	59 (10.6)	2.61E-04
>20	100	58	92 (17.5)	53 (9.5)	5.32E-04

^#^Housekeeping genes, *Tissue specific genes. A total of 525 housekeeping and 558 tissue specific genes were analysed. The numbers in parentheses (4^th ^& 5^th ^columns) represent the percentage of genes containing the repeat stretch.

^§^Difference in the distribution of poly (dA.dT) stretches in Hkg and Tsg analysed by applying *t*-test (for normalizing the difference in sample size). The repeat lengths from >12 to >18 bp are showing very significantly different distributions between Hkg and Tsg. The distributions were examined in 2000 bp upstream region from the gene start site.

**Table 3:** *t*-test *P*-values for the difference in the distribution of nucleosome formation potential scores between housekeeping and tissue specific genes.

Length (bp)*	***P*-value in intervals of scores**			
	
	**-1.2 to -1**	**-1 to -0.8**	**0.8 to 1**	**1 to 1.2**
400	3.53E-13	8.73E-17	1.28E-17	4.45E-23
800	1.16E-24	6.27E-24	6.64E-12	6.65E-22
1200	6.91E-26	1.44E-24	2.10E-09	1.64E-18
1600	1.72E-24	6.99E-24	2.72E-07	5.63E-15
2000	2.55E-25	1.84E-25	3.22E-05	6.71E-13

*denotes the length of 5' upstream region from the gene start site taken for the analysis. The scores were compared in the four Recon score intervals of relevance -1.2 to -1, -1 to -0.8, 0.8 to 1 and 1 to 1.2.

**Table 4:** Correlation coefficients of total expression levels (log_10_) with nucleosome formation potential scores in housekeeping (Hkg) and tissue specific genes (Tsg).

**Category**	**Correlation coefficient**			
	
	**-1.2 to -1**	**-1 to -0.8**	**0.8 to 1**	**1 to 1.2**
Hkg^#^	0.10	0.14	0.04	-0.01
Tsg*	-0.10	-0.12	0.15	0.17

^#^Housekeeping genes, *Tissue specific genes.

The correlation was drawn in the four Recon score intervals of relevance -1.2 to -1, -1 to -0.8, 0.8 to 1 and 1 to 1.2.

**Table 5:** Comparison of the level of correlation between nucleosome formation potential scores and contrasting expression levels of genes.

*Category	**Correlation coefficient**			
	
	**-1.2 to -1**^#^	**-1 to -0.8**^#^	**0.8 to 1**^#^	**1 to 1.2**^#^
Hkg ↑↑	0.17	0.30	-0.10	-0.26
Hkg ↓↓	0.36	0.35	-0.28	-0.39
Tsg ↑↑	0.03	0.02	0.26	0.11
Tsg ↓↓	-0.12	-0.14	0.15	0.16

*High and low expression level genes were categorised in Hkg (housekeeping genes) and Tsg (tissue specific genes) groups separately. ^# ^Recon score intervals. The genes classified as high and low expression genes in both Hkg and Tsg had atleast a 10-fold difference in their expression levels. The up (↑↑) and down (↓↓) arrows denote high expression and low expression respectively.

**Table 6:** The distribution of Alu repeats in 5' upstream regions of housekeeping (Hkg) and tissue specific genes (Tsg) is represented in terms of the number of copies and basepairs covered by Alu repeats.

**Repeat category**	**No. of copies**		**% of the total sequences covered by the repeat**	
	
	**Hkg**^#^	Tsg*	**Hkg**^#^	Tsg*
Alu	866	575	20.1	12.3

^#^Housekeeping genes, *Tissue specific genes.

**Table 7:** *t*-test *P*-values for the difference in the distribution of Alu repeats in 5' upstream regions of housekeeping and tissue specific genes.

**Repeat Category**	***P*-value**	
	
	**No. of repeats**	**Repeat content (bp)**
Alu	3.02E-08	7.63E-11
